# Complete Genome Sequence of *Staphylococcus carnosus* LTH 3730

**DOI:** 10.1128/genomeA.01038-16

**Published:** 2016-09-29

**Authors:** Anne Müller, Jochen Klumpp, Herbert Schmidt, Agnes Weiss

**Affiliations:** a Department of Food Microbiology and Hygiene, Institute of Food Science and Biotechnology, University of Hohenheim, Stuttgart, Germany; b Institute of Food, Nutrition and Health, ETH Zurich, Zurich, Switzerland

## Abstract

Specific strains of the apathogenic coagulase-negative species *Staphylococcus carnosus* are frequently used as meat starter cultures, as they contribute to color formation and the production of aroma compounds. Here, we report the complete genome sequence of *S. carnosus* LTH 3730, a strain isolated from a fermented fish product.

## GENOME ANNOUNCEMENT

*Staphylococcus carnosus* is often used as a starter culture for fermented meat products, such as fermented sausage and cured raw ham. Due to different enzymatic activities, specific *S. carnosus* strains contribute to color formation (1) and the production of different aroma compounds (2). The species *S. carnosus* can be divided into two subspecies, *S. carnosus* subsp. *carnosus* and *S. carnosus* subsp. *utilis*, but strain LTH 3730 could not be assigned to one of those in a former study (3). LTH 3730 was originally isolated from Pla-chom, a fermented fish product of Thailand (4) (alternative name, SK13 [5]) and was confirmed as *S. carnosus* by sequencing of the 16S rRNA gene and *sodA* (3). Genomic DNA of *S. carnosus* LTH 3730 was isolated from a 2-ml overnight culture (brain heart infusion [BHI] broth at 37°C) using the GenElute bacterial genomic DNA kit (catalog no. NA2100; Sigma-Aldrich Chemie GmbH, Munich, Germany), according to the protocol for Gram-positive bacteria. The optional RNase A step was included in the preparation, and the elution step was executed twice. Two independent DNA isolations were combined for sequencing.

The bacterial chromosome was sequenced on a PacBio RSII device (Pacific Biosciences, Menlo Park, CA), with a 10-kb size-selected insert library and P6/C4 chemistry. The sample was sequenced on two single-molecule real-time (SMRT) cells. *De novo* assembly (HGAP3 algorithm) was performed using SMRT Analysis version 2.3 (Pacific Biosciences). HGAP3 settings were kept at the defaults, except for the expected genome size, which was set at 2.5 Mbp. A total of 48,794 reads with 666 Mbp total bases and a mean read length of 13,656 bp were produced. The HGAP3 analysis produced a complete *de novo*-assembled genome sequence with >200-fold coverage over the entire molecule.

Redundant ends of the sequence were identified by creating a dot plot of the sequence against itself in UGENE version 1.13.2 (6). One redundant end was cut, and the result was confirmed by PCR and Sanger sequencing.

The complete genome of LTH 3730 has a size of 2,645,106 bp and a G+C content of 34.6%. RAST (7) predicted 2,579 coding sequences. Two intact prophages, which have sizes of 49.5 kb and 51.3 kb, respectively, were identified with PHAST (8).

A total of 26 genes involved in oxidative stress response were detected in LTH 3730, including *sodA*, *sodB*, *sodC*, and genes encoding catalase and peroxidases. Similar to the already published *S. carnosus* genomes, the complete pathway for the reduction from nitrate to nitrite and further on to ammonia was found. This characteristic is important for a starter culture, as this can contribute to color development. However, LTH 3730 was excluded as a starter culture in a former study, as it showed hemolytic activity (9). The genome sequence was therefore analyzed with PathogenFinder 1.1 (10), and several hypothetical proteins were found to match pathogenic staphylococci, although none of them were identified as a hemolysin.

### Accession number(s).

This complete genome project has been deposited in GenBank under the accession no. CP016760. The version described in this paper is the first version.
